# Association between Serum Atypical Fibroblast Growth Factors 21 and 19 and Pediatric Nonalcoholic Fatty Liver Disease

**DOI:** 10.1371/journal.pone.0067160

**Published:** 2013-06-26

**Authors:** Anna Alisi, Sara Ceccarelli, Nadia Panera, Federica Prono, Stefania Petrini, Cristiano De Stefanis, Marco Pezzullo, Alberto Tozzi, Alberto Villani, Giorgio Bedogni, Valerio Nobili

**Affiliations:** 1 Hepato-Metabolic Disease Unit and Liver Research Unit, Bambino Gesù Children’s Hospital, IRCCS, Rome, Italy; 2 Microscopy Unit, Bambino Gesù Children’s Hospital, IRCCS, Rome, Italy; 3 Core Facilities, Bambino Gesù Children’s Hospital, IRCCS, Rome, Italy; 4 Epidemiology Unit, Bambino Gesù Children’s Hospital, IRCCS, Rome, Italy; 5 Pediatrics and Infectious Disease Unit, Bambino Gesù Children’s Hospital, IRCCS, Rome, Italy; 6 Clinical Epidemiology Unit, Liver Research Center, Trieste, Italy; The University of Hong Kong, Hong Kong

## Abstract

Atypical fibroblast growth factors (FGF) 21 and 19 play a central role in energy metabolism through the mediation of Klotho coreceptor. Contradictory findings are available about the association of FGF21 and FGF19 with nonalcoholic fatty liver disease (NAFLD) in humans. We investigated the association of serum FGF21, FGF19 and liver Klotho coreceptor with non-alcoholic steatohepatitis (NASH) and fibrosis in children with NAFLD. Serum FGF21 and FGF19 were measured in 84 children with biopsy-proven NAFLD and 23 controls (CTRL). The hepatic expression of Klotho coreceptor was measured in 7 CTRL, 9 patients with NASH (NASH+) and 11 patients without NASH (NASH−). FGF21 and FGF19 showed a tendency to decrease from CTRL (median FGF21 = 196 pg/mL; median FGF19 = 201 pg/mL) to NASH− (FGF21 = 89 pg/mL; FGF19 = 81 pg/mL) to NASH+ patients (FGF21 = 54 pg/mL; FGF19 = 41 pg/mL) (*p*<0.001 for all comparisons) and were inversely associated with the probability of NASH and fibrosis in children with NAFLD. The hepatic expression of Klotho coreceptor was inversely associated with NASH (R^2^ = 0.87, p<0.0001) and directly associated with serum FGF21 (R^2^ = 0.57, p<0.0001) and FGF19 (R^2^ = 0.67, p<0.0001). In conclusion, serum FGF19 and FGF21 and hepatic Klotho expression are inversely associated with hepatic damage in children with NAFLD and these findings may have important implications for understanding the mechanisms of NAFLD progression.

## Introduction

The prevalence of non-alcoholic fatty liver disease (NAFLD), which is considered the hepatic manifestation of the metabolic syndrome (MS), is rapidly increasing worldwide [1]. NAFLD ranges from simple liver steatosis to nonalcoholic steatohepatitis (NASH), which is a predictor of fibrosis and end-stage liver disease [2]. Although obesity and selected components of the MS are known risk factors for NALFD [3], the mechanisms by which these factors produce the large spectrum of disease typical of NAFLD are largely unknown [4], [5].

In this respect, it is very interesting that fibroblast growth-factor 21 (FGF21), an autophagy-regulated mitokine, has been shown to play a central role in the maintenance of glucose and lipid homeostasis [6]. Autophagy-deficient mice have up-regulated FGF-21 levels, increased beta-oxidation in muscle and adipose tissue, reduced fat mass, heightened insulin sensitivity, and they are spared from diet-induced obesity and hepatosteatosis. This evidence suggests that FGF21 and its networked molecular environment is a supervisor of lipid accumulation in adipose tissue and other peripheral organs such as the liver [7].

FGF21 belongs to the family of atypical FGFs, which include FGF15 and FGF19 (mouse and human orthologs, respectively) and FGF23 (which lacks the conventional FGF heparin-binding domain). Under specific conditions, FGFs can be released into the circulation and act as paracrine and endocrine factors [8]. The binding of FGF family members to their specific receptors (FGFRs) activate signaling cascades that participate to the control of cell-to-cell communication, regulation of development, and cellular homeostasis [9]. The stability of FGF binding to FGFRs with consequent intracellular signaling require single-pass transmembrane proteins called Klotho and β-Klotho coreceptors. The tissue-specific expression of Klotho and β-Klotho determines the target organs of the atypical FGFs [10]. Although the cellular origin of FGF21 and FGF19 and the regulation of their expression are not well understood, FGF19 is known to be expressed in the intestine and plays important roles in regulating bile acid synthesis, phosphate homeostasis and enterohepatic signaling [11], [12]. FGF21 is expressed in liver, pancreas, skeletal muscle and adipose tissue and it is a crucial regulator of glucose and lipid homeostasis [18], [19]. Interestingly, administration of either FGF21 or FGF19 has beneficial effects on lipid metabolism and improves hepatic steatosis in animal models with diet-induced obesity [13]–[15].

A cross-sectional study performed in adults with abnormal glucose metabolism showed a direct association of serum FGF21 with liver fat measured by magnetic resonance spectroscopy [16]. However, lower serum FGF21 levels were found in patients with severe hepatosteatosis and it was speculated that this might due to associated lipotoxicity- and necro-inflammation [16]. More recently, lower serum levels of FGF19 were reported in adult NAFLD independently of the severity of liver damage [17].

Despite many experimental findings suggest that FGF21 and FGF19 are promising anti-hepatosteatotic agents, much remains to be known about their association with liver disease in humans [18]. Therefore, the aim of the present cross-sectional study was to explore the FGFs/Klotho-NAFLD severity association in a series of children with NAFLD followed at a pediatric Hepatology Center.

## Subjects and Methods

### Study Design

Eighty-four children (32 F and 52 M) with biopsy-proven NAFLD and 23 controls (CTRL, 9 F and 14 M) without evidence of fatty liver at ultrasonography were consecutively enrolled into the study at the Hepatology Unit of the Bambino Gesù Children’s Hospital between May 2010 and December 2011. Inclusion criteria were: 1) complete abstinence from alcohol, 2) absence of serological markers of hepatitis B and C, 3) absence of drugs known to induce fatty liver and, 4) ceruloplasmin, anti-transglutaminase antibodies, antinuclear antibodies, anti-mitochondrial antibodies and anti-smooth muscle antibodies within normal limits. The study protocol was approved by the Ethical Committee of the Bambino Gesù Children’s Hospital and written informed consent was obtained from the parents of the children.

### Anthropometry

Weight and height were measured using standard procedures [19]. Body mass index (BMI) was calculated as weight (kg)/height (m)^2^ and transformed into standard deviations scores (SDS) using Italian reference values [20].

### Laboratory Measurements

Alanine transaminase (ALT), aspartate transaminase (AST), gamma-glutamyl-transferase (GGT), glucose, triglycerides and cholesterol were measured by standard laboratory methods. All measurements were performed at 7∶00 AM after an overnight fasting. For FGF21 and FGF19 measurements, the blood centrifuged at 8000 RPM for 12 min. and stored at −80°C pending further analysis. Samples were thawed only once and measured using ELISA (BioVendor, Modřice, Czech Republic).

### Liver Histopathology

Liver biopsy was performed as described in detail elsewhere [21]. Bioptic specimens were fixed in 10% buffered formalin pending further analysis. NAFLD was diagnosed and staged using the NAFLD Clinical Research Network criteria [22]. All bioptic samples were examined by the same experienced pathologist.

### Immunofluorescence

Liver tissue from 9 NASH+, 11 NASH− patients, and 7 controls (CTRL) were used to perform immunofluorescent staining for Klotho coreceptor, alpha-smooth muscle actin (alpha-SMA), cytokeratin 8/18 (CK8/CK18) and CD163. *CTRL patients were not related to the present study but had undergone a liver biopsy showing no liver steatosis during surgical treatment for acute appendicitis.* OCT-embedded liver tissue was sectioned with a cryostat (5 µm) and fixed *in ice-cold acetone. Tissue slides were then blocked in 5% BSA for 1 hr and incubated with: 1∶400 anti-Klotho rabbit polyclonal antibody (Alpha Diagnostic International, San Antonio, TX, USA) overnight at 4°C; 1∶200 anti-CD163 mouse monoclonal antibody (*Novocastra, New Castle, UK) *overnight at 4°C; 1∶100 anti-CK8/CK18 mouse monoclonal antibody (Vector* Laboratories Inc., California, USA*) for 1 hr at room temperature; 1∶200 anti-alpha-SMA mouse monoclonal antibody (Novus Biological, Littleton, CO, USA) 1 hr at room temperature.*

Detection of the primary antibodies was performed using 1∶500 Alexa Fluor 488 goat anti-rabbit IgG and Alexa Fluor 555 goat anti-mouse IgG secondary antibodies (Invitrogen/Molecular Probes Corp, *Carlsbad, CA* USA) for 1 *hr at room temperature*. For nuclear staining, 4′,6-diamidino-2-phenylindole was added for 5 min. before section mounting with glycerol/PBS (1/1). Confocal imaging was performed using an Olympus Fluoview FV1000 confocal microscope equipped with FV10-ASW 2.0 software, Multi Ar (458–488 and 515 nm), 2× He/Ne (543 and 633 nm), 405-nm diode lasers and a 60× (numerical aperture 1.42) oil objective. Optical sections were acquired with a resolution of 1024×1024 pixels, a sampling speed of 40 µs/pixel, and 12 bits/pixel images. Fluorochrome unmixing was performed by automated-sequential collection of multi-channel images to reduce spectral cross-talk between channels. Negative control was performed omitting primary antibodies (see **Figure S1**). As positive control Klotho was stained on gut and kidney samples (see **Figure S2**).

For quantification of *Klotho coreceptor* hepatic expression of *t*he free area from single section profile was manually used to draw the region of interest (ROI). The fluorescence intensity was measured by Olympus FVIO-ASW 2.0 software from at least 50 ROI taken from three randomly selected images from each sample. The average fluorescence intensity was calculated using Excel (Microsoft, US).

### Statistical Analysis

Most continuous variables were not normally distributed and all are reported as 50^th^, 25^th^ and 75^th^ percentiles. Categorical variables are reported as numbers and percentages. Between-group comparisons of medians were performed using quantile regression [23]. When more than two groups were involved in between-group comparisons, Bonferroni’s correction was employed. Logistic regression was used to evaluate whether FGF21 (continuous, pg/mL) and FGF19 (continuous, pg/mL) were associated with NASH (0 = no; 1 = yes) and with any degree of fibrosis (discrete, 0 = no; 1 = yes). In addition to univariable models, we used multivariable models including age (continuous, years), gender (discrete, 0 = female; 1 = male) and BMI (continuous, SDS) as predictors together with FGF19 or FGF21, to test whether the relationships of interest was influenced by these potential confounders. Fractional polynomials showed that a log-transformation of FGF19 and FG21 was needed to ensure a linear logit for all outcomes [24], [25]. Akaike information criterion (AIC) was used to evaluate model fit [26]. Linear regression was used to evaluate the degree of association between the log-transformed values of the Klotho receptor (log_e_ Klotho), the histopathological status (discrete, 0 = control; 1 = NASH negative; 2 = NASH positive) and log_e_FGF21 (continuous) and log_e_FGF19 (continuous). Statistical analysis was performed using Stata version 12.1 (Stata Corp, College Station, TX, USA).

## Results

### Comparison of Controls and Children with NAFLD, with NASH and without NASH

**Table 1** reports the 50^th^, 25^th^ and 75^th^ percentiles of age, anthropometry, serum FGF21and FGF19 and other laboratory parameters in controls (n = 23) and children (n = 84) with NAFLD. The median levels of FGF21 and FGF19 were significantly lower in NAFLD patients with respect to controls (p<0.001, quantile regression).

**Table 1:** Measurements of control and NAFLD children.

	Controls			NAFLD			P50
	(*n* = 23)			(*n* = 84)			comparison*
	P50	P25	P75	P50	P25	P75	p-value
*Age (years)*	11	4	15	10	9	11	0.3
*Weight (Kg)*	55	36	65	53	41	62	0.5
*Height (m)*	1.41	1.16	1.56	1.45	1.33	1.53	0.6
*BMI (kg/m^2^)*	25.5	24.5	27.2	25	22.2	27.4	0.6
*BMI (SDS)*	2.19	1.34	2.56	1.74	1.41	2.04	<0.001
*ALT (U/L)*	30	26	34	70	48	90	<0.001
*AST (U/L)*	29	26	30	51	40	65	<0.001
*GGT (U/L)*	20	19	23	24	17	34	0.2
*Glucose (mg/dL)*	80	78	82	81	75	89	0.3
*Triglycerides (mg/dL)*	84	78	90	91	75	124	0.2
*Cholesterol (mg/dL)*	128	123	130	160	132	190	<0.001
*FGF21 (pg/mL)*	196	176	222	77	58	96	<0.001
*FGF19 (pg/mL)*	201	190	247	70	48	89	<0.001

* Median (P50) comparison performed with quantile regression.

Abbreviations: P = percentile; BMI = body mass index; ALT = alanine transaminase; AST = aspartate transaminase; GTT = gamma-glutamyl-transferase; FGF = fibroblast growth factor.

The histopathological features of the 84 children with NAFLD are given in **Table 2**. As diagnosed by the liver pathologist, 32 (38%) of NAFLD children had NASH and fibrosis of any degree was present in 59 (70%) of cases.

**Table 2:** Liver histopathology of the 84 children with NAFLD.

	N	%
***Steatosis***		
0	1	1.2
1	21	25
2	40	47.6
3	22	26.2
***Inflammation***		
0	7	8.3
1	65	77.4
2	12	14.3
***Ballooning***		
0	40	47.6
1	27	32.1
2	17	20.2
***NAS***		
1	2	2.4
2	18	21.4
3	21	25
4	14	16.7
5	16	19
6	11	13.1
7	2	2.4
***NASH***		
No	52	61.9
Yes	32	38.1
***Fibrosis***		
0	25	29.8
1	47	56
2	5	6
3	7	8.3

Abbreviations: NAS = NAFLD Activity Score.

**Figure 1:**
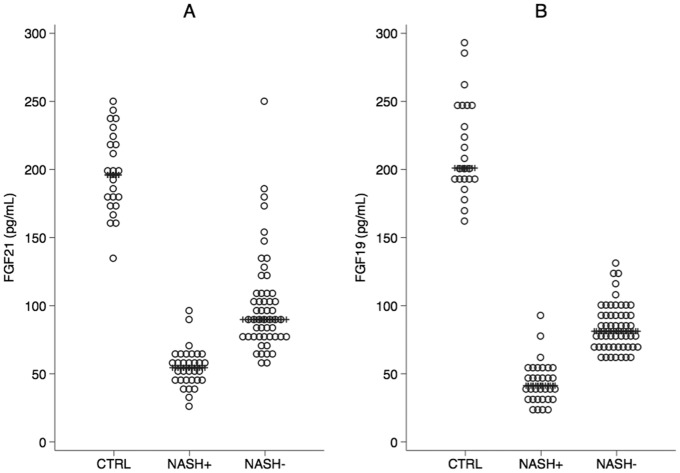
NASH and serum levels of FGF21 and FGF19. Distribution of serum FGF21 (panel **A**) and FGF19 (panel **B**) in controls, NAFLD children without NASH (NASH−) and NASH children with NASH (NASH+). Lines superimposed to dot-plots are medians.

Interestingly, the median (25^th^; 75^th^ percentile) values of FGF21 were 196 (176;222) in controls, 54 (45;63) in children with NASH (NASH+) and 89 (78;100) in those without NASH (NASH−) children and the corresponding values for FGF19 were 201 (190;247), 41 (33;50) and 81 (70;96) (**Figure 1** **, panel A**). The median values of FGF19 and FGF21 were significantly lower in NASH− and NASH+ children *vs.* controls and lower in NASH+ children compared to NASH− children (*p*<0.001 for all comparisons, quantile regression with Bonferroni’s correction) (**Figure 1** **, panel B**).

### Association between Serum FGFs, NASH and Fibrosis

**Figure 2:**
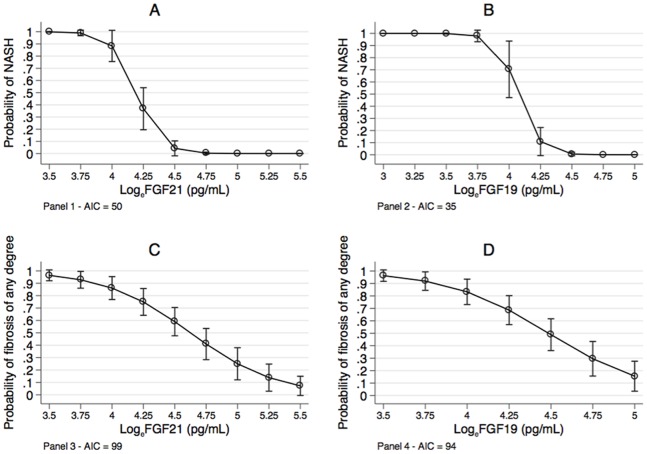
Probability of NASH and fibrosis as a function of serum FGF21 and FGF19. Probability of NASH and fibrosis of any degree as a function of log_e_-transformed values of FGF21 and FGF19 (see text for further details on statistical analysis). Abbreviations: NASH = non-alcoholic steatohepatitis; log_e_ = natural logarithm; AIC = Akaike information criterion. Circles are means and bars 95% confidence intervals.

**Panel A** of **Figure 2** plots the probability of NASH estimated by logistic regression as a function of log_e_FGF21. Increasing values of log_e_FGF21 are associated with a lower probability of NASH (*p*<0.001). The log-odds (standard error, SE) of log_e_FGF21 was −10.6 (2.5) (*p*<0.001) and did not change when age [continuous, 0.1 (0.2)], male gender [yes *vs.* no, 0.5 (0.9)] and BMI [continuous, SDS, 0.1 (0.5)] were added to the model (*p*>0.05 for all). Thus, there was no evidence of confounding of the NASH−log_e_FGF21 relationship from age, gender or BMI.

**Panel B** of **Figure 2** plots the probability of NASH as a function of log_e_FGF19. As for FGF21, increasing values of log_e_FGF19 are associated with a lower probability of NASH (*p*<0.001). The log-odds (SE) of log_e_FGF19 was −12.0 (3.0) (*p*<0.001) and did not change when age [0.0 (0.2)], male gender [−0.2 (1.0)] and BMI [0.3 (0.7)] were added to the model (*p*>0.05 for all). Thus, there was no evidence of confounding of the NASH−log_e_FGF19 relationship from age, gender or BMI.

Log_e_FGF21 and log_e_FGF19 were associated not only with binary NASH but also with continuous NAS. The association was linear in both cases, with NAS explaining 28% of log_e_FGF21 variance and 25% of log_e_FGF19 variance (*p*<0.001 for both). According to these regression models, an increase of 1 unit of NAS is associated to a mean (SE) decrease of log_e_FGF21 equal to −0.13 (0.03) log-units and a mean (SE) decrease of Log_e_FGF19 equal to −0.14 (0.03) log-units. The relationship was unchanged when age (continuous), gender (male *vs.* female) and BMI (SDS) were added to the regression models (*p*>0.05 for all). Thus, there was no evidence of confounding of the NAS-log_e_FGF21 and NAS-log_e_FGF19 relationships attributable to age, gender or BMI.

**Panel C** of **Figure 2** plots the probability of fibrosis of any degree as a function of log_e_FGF21. Increasing values of log_e_FGF21 are associated with a lower probability of fibrosis (*p*<0.001). The log-odds (SE) of log_e_FGF21 was −2.0 (0.8) (*p*<0.001) and did not change when age [0.2 (0.1)], male gender [−0.2 (0.5)] and BMI [0.6 (0.4)] were added to the model (*p*<0.05 for all). Thus, there was no evidence of confounding of the fibrosis-log_e_FGF21 relationship from age, gender or BMI.

**Panel** **D** of **Figure 2** plots the probability of fibrosis of any degree as a function of log_e_FGF19. As for FGF21, increasing values of log_e_FGF19 are associated with a lower probability of fibrosis (*p*<0.001). The log-odds (SE) of log_e_FGF21 was −2.6 (0.9) (*p*<0.001) and did not change when age [0.2 (0.1)], male gender [−0.2 (0.5)] and BMI [0.6 (0.4)] were added to the model (*p*>0.05 for all). Thus, there was no evidence of confounding of the fibrosis-log_e_FGF19 relationship from age, gender or BMI.

It is of some interest that NASH was more strongly associated with log_e_FGF19 than with log_e_FGF21 (AIC = 35 *vs.* AIC = 50). Likewise, fibrosis was more strongly associated with log_e_FGF19 than with log_e_FGF21 (AIC = 94 *vs.* AIC = 99).

### Association of Liver Klotho coreceptor with Liver Histology and Serum FGFs

Although the binding of β-Klotho coreceptor is central for the action of FGF21 and FGF19 in the liver [16], [27], hepatic Klotho coreceptor expression is crucial for autophagy [28], which in turn may regulate at least FGF21 production and release [6]. There are no evidence about Klotho hepatic expression in NAFLD and its potential correlation with FGF21 and FGF19.

Therefore, here we measured Klotho hepatic expression in a randomly chosen subsample of 20 NAFLD children (11 NASH− and 9 NASH+). Seven children unrelated to the present study, who underwent urgent appendectomy and had liver bioptic specimens without signs of steatosis, provided the control group for this comparison. **Table 3** compares of CTRL, NASH− and NASH+ children. Besides the expected decrease of serum FGF21 and FGF19, there was a decrease of hepatic Klotho expression with increasing severity of NAFLD.

**Table 3:** Measurements of children evaluated in the Klotho substudy.

	Controls			NASH−			NASH+		
	(*n* = 7)			(*n* = 11)			(*n* = 9)		
	p50	p25	p75	p50	p25	p75	p50	p25	p75
*Age (years)*	11^a^	4	16	10^a^	10.1	12	11^a^	10	11
*Weight (Kg)*	48.0^a^	26	64	50.0^a^	43	66	50.0^a^	42	60
*Height (m)*	1.40^a^	1	1.6	1.50^a^	1.4	1.5	1.40^a^	1.3	1.5
*BMI (kg/m^2^)*	25.5^a^	24.4	25.9	25.5^a^	22.6	27.3	23.1^a^	21.9	26.9
*BMI (SDS)*	1.60^a^	1.1	2.5	1.70^a^	1.3	2.3	1.40^a^	1.3	2.2
*ALT (U/L)*	28^a^	23	32	66^b^	39	80	85^c^	69	107
*AST (U/L)*	29^a^	29	31	39^b^	32	56	56^b^	53	57
*GGT (U/L)*	24^a^	22	31	25^a^	17	27	27^a^	21	34
*Glucose (mg/dL)*	79^a^	78	82	84^a^	72	90	76^a^	75	80
*Triglycerides (mg/dL)*	80^a^	74	85	82^a^	72	112	99^a^	74	170
*Cholesterol (mg/dL)*	128^a^	126	131	133^a^	127	167	150^b^	129	188
*FGF21 (pg/mL)*	178^a^	172	217	101^b^	67	123	61^c^	50	67
*FGF19 (pg/mL)*	244^a^	190	249	80^b^	72	100	41^c^	30	56
*Klotho (mean of FI)*	1225^a^	1096	1312	805^b^	680	898	425^c^	364	497

Medians not sharing the same superscript are significantly different at a level of *p*<0.05 (quantile regression with Bonferroni’s correction).

Abbreviations: P = percentile; BMI = body mass index; ALT = alanine transaminase; AST = aspartate transaminase; GTT = gamma-glutamyl-transferase; FGF = fibroblast growth factor; FI = fluorescence intensity.

**Figure 3:**
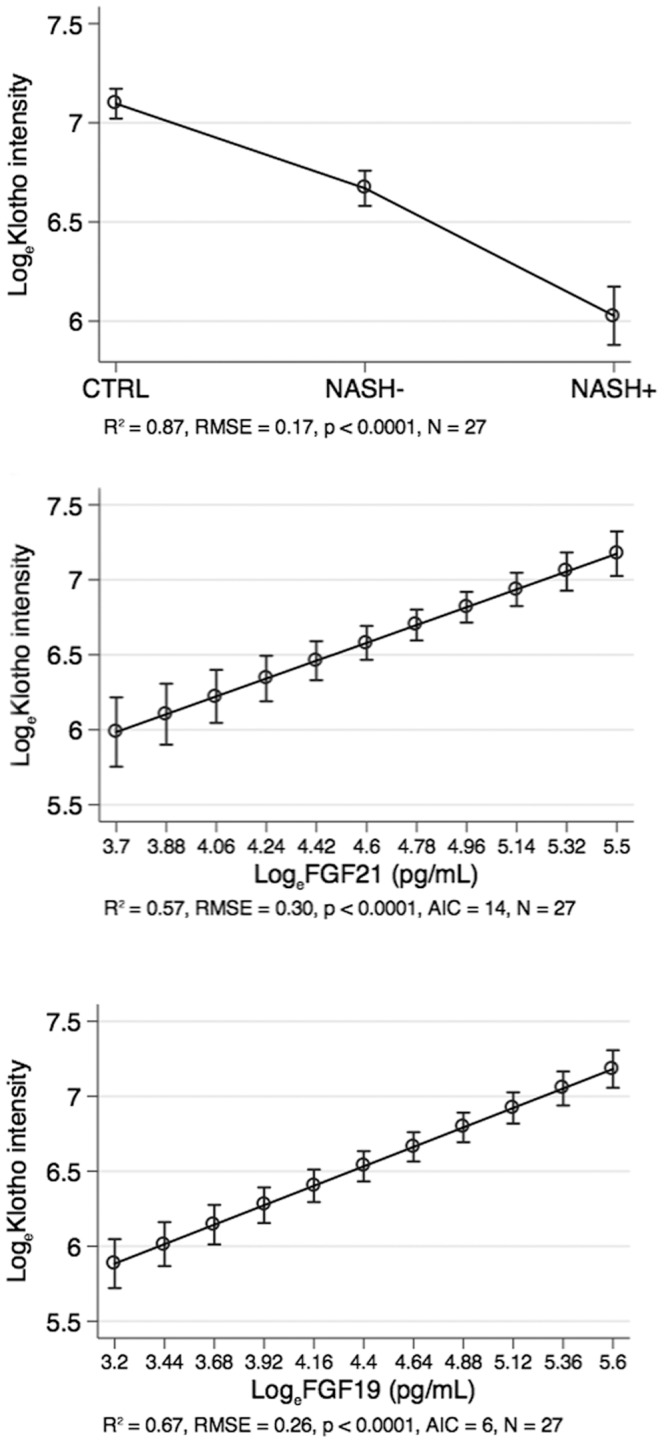
Association between the hepatic expression of Klotho, liver histopathology and serum FGF21 and FGF19. Association of Klotho with histopathology, FGF21 and FGF19 (see text for further details on statistical analysis). Abbreviations: log_e_ = natural logarithm; RMSE = root mean squared error of the estimate; R^2^ = coefficient of determination; AIC = Akaike information criterion. Circles are means and bars 95% confidence intervals.

As shown in **Figure 3**, the hepatic expression of Klotho coreceptor was lower in NASH+ than in NASH− and CTRL children and NASH status (discrete; 0 = CTRL, 1 = NASH−, 2 = NASH+) was able to explain 87% of the variance of Log_e_Klotho (*p*<0.0001).

On the other hand, log_e_FGF21 and log_e_FGF19 were directly associated with Log_e_Klotho and explained respectively 57 and 67% of its variance (*p*<0.0001). Log_e_Klotho was more strongly associated with Log_e_FGF19 than with log_e_FGF21 (AIC = 6 *vs*. AIC = 14).

**Figure 4:**
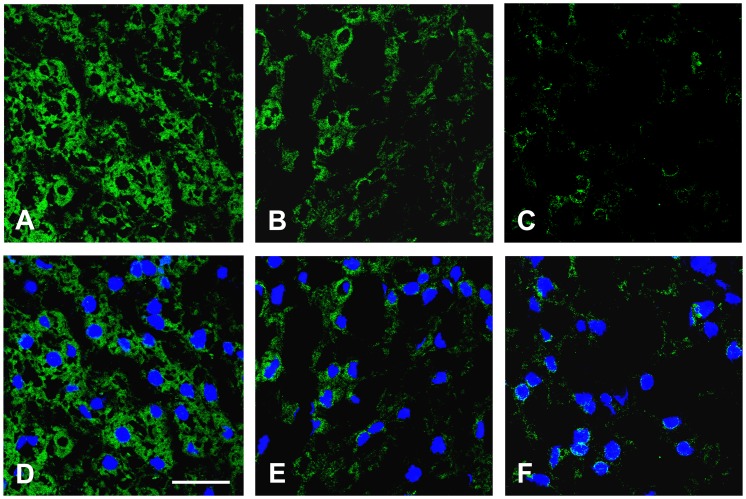
Representative confocal immunofluorescence for expression and intracellular distribution of Klotho co-receptor. The representative confocal immunofluorescence was performed on liver tissue cryostat sections OCT-embedded. The staining of Klotho co-receptor in the overweight-obese children without NAFLD (**A**), in the NAFLD overweight-obese children without NASH (**B**), and in the overweight-obese children with NASH (C) is shown in green. The nuclei are revealed by specific DAPI staining, displayed in blue. The white bar represents a 30 µm length.

As shown in **Figure 4**, the hepatic Klotho coreceptor was localized mainly in the cytoplasmic compartment in CTRL, whereas it was localized in the perinuclear region of liver-resident cells in NASH− and NASH+ subjects.

**Figure 5:**
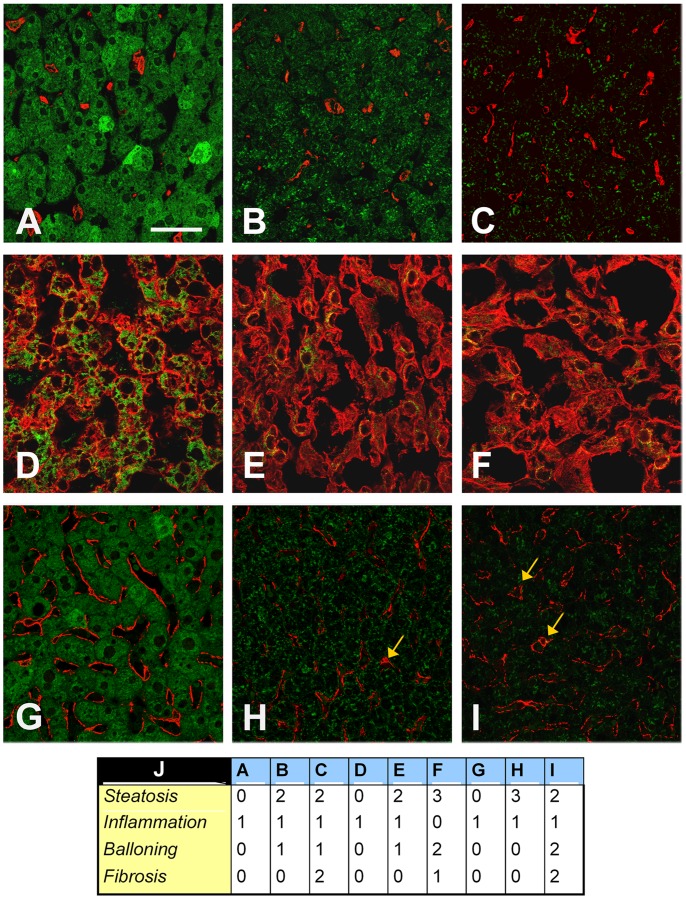
Representative confocal immunofluorescence of Klotho co-receptor localization in liver tissues. The representative confocal immunofluorescence was performed on liver tissue cryostat sections OCT-embedded. (**A–C**) Co-staining of Klotho co-receptor (green) and CD163 (red) in children without NAFLD (**A**), with NAFLD NASH− (**B**) and with NASH+ (**C**). (**D–F**) Co-staining of Klotho co-receptor (green) and cytokeratin 8/18 (red) in children without NAFLD (**D**), with NAFLD NASH− (**E**) and with NASH+ (**F**). (**G–I**) Co-staining of Klotho co-receptor (green) and alpha-SMA (red) in children without NAFLD (**G**), with NAFLD NASH− (**H**) and with NASH+ (**I**). Yellow arrows indicate hepatic stellate cells. The white bar represents a 30 µm length. (**J**) Histological scores for panels from A to I.

To evaluate which liver-resident cells expressed Klotho coreceptor we performed a co-staining with specific markers of endothelial and hepatic stellate cells (alpha-SMA), hepatocytes (cytokeratin 8/18) and macrophages/Kupffer cells (CD163). As shown in **Figure 5** **(D–F)**, Klotho coreceptor was expressed only in hepatocytes.

## Discussion

To our knowledge, this is the first comprehensive study to evaluate the association between FGF21, FGF19 and NAFLD severity. FGF21 and FGF19 were lower in NAFLD than in CTRL children and were inversely associated with the probability of NASH and fibrosis. Moreover, the hepatic expression of Klotho coreceptor was a predictor of NASH and was directly associated with the circulating levels FGF21 and FGF19.

Our study has some limitations. First, children were recruited at an Hepatology Clinic so that our “case-mix” of patients is not comparable to that of the Obesity Clinics where the majority of studies of FGFs and fatty liver have been performed to date [29]. Second, the sample size for the study of the Klotho coreceptor was about 1/4^th^ of that of the whole study (n = 27 *vs.* n = 107). In spite of the strength of Klotho-NASH and Klotho-FGF relationships, the small sample sizes might misrepresent the real findings that should to be replicated in larger external samples. Moreover, although NASH+ (*n* = 9) and NASH− (*n* = 11) children for the Klotho sub-study were randomly chosen from the main study, for obvious ethical reasons, control children (*n* = 7) were not. These controls are of course “imperfect” because of signs of hepatic necro-inflammation, as demonstrated by the presence of CD163-positive cells similar NASH+ samples, but their hepatocyte expression of Klotho co-receptor remains statistically higher than NAFLD and NASH (Table 3).

Reinehr and colleagues detected higher values of FGF21 in obese than in normal-weight children but they found no association between FGF21 and NAFLD [29]. They diagnosed NAFLD on the basis of ultrasonography while we used liver biopsy to diagnose and stage NAFLD [30]. This allowed us to better disentangle the NALFD-FGFs relationship by separating simple steatosis from NASH and by separately evaluating fibrosis, which is clearly the “hardest” hepatological outcome for pediatric NAFLD [1]. Yan and colleagues reported lower FGF21 levels in severe steatosis as evaluated by magnetic resonance imaging [16]. In the present work, we focused on NASH and fibrosis because of their prognostic significance [31]. Our finding of a strong inverse association between FGF21 and NAFLD severity is supported by two recent experimental studies showing that tumor necrosis factor and oxidative stress-activated transcription factors, such as NFE2-related factor 2, may impair FGF21 transcription and release [32], [33].

An inverse association between serum FGF19 and the presence of MS has been reported [34] and insulin-resistant patients with NAFLD have been shown to exhibit an impaired hepatic response to FGF19 [35]. Moreover, reduced levels of FGF19 have been reported in patients with biopsy-proven NAFLD [36] and the present study confirms that FGF19 is inversely associated to NAFLD severity in terms of both NASH and fibrosis. It is of some interest that the association with NAFLD severity was stronger for FGF19 than for FGF21 both in terms of NASH and fibrosis, even if FGF19 and FGF21 were expectedly strictly associated (data not shown). This finding may be relevant for the comprehension of the mechanisms of progression of NAFLD to HCC. In fact, differently from FGF21, FGF19 is mitogenic for hepatocytes and its signaling through the FGFR4/β-klotho complex is frequently up-regulated in human HCC tissues [37].

We found a strong inverse association between hepatic Klotho coreceptor activation and NASH and a strong direct association of hepatic Klotho coreceptor and serum FGF21 and FGF19. Although the β-Klotho coreceptor is crucial for FGF21 and FGF19 signaling [38], [39], little is known about Klotho hepatic expression during NAFLD. A recent study showed that the hepatic Klotho expression correlated with cirrhosis (stage 4 fibrosis) in HCC, demonstrating a novel pro-oncogenic function of this protein [40]. We found Klotho protein in the cytoplasmic and perinuclear region of hepatocytes in controls and a progressive decrease of its cytoplasmic expression occurred in NAFLD. The total absence of Klotho on plasma membrane of hepatocyte is in agreement with its specific binding to FGF23 in other organs, such as kidney [41]. However, as we used an antibody recognizing an aminoacidic sequence within the C-terminal cytoplasmic domain of Klotho (see **Figure S3**) we cannot establish if the hepatic Klotho is complete or contains only the cytoplasmic region obtained after the metalloproteases-dependent cleavage of secreted form [41]. This information could be critical to understand the role of Klotho coreceptor in liver pathophysiology.

It remains to be defined why serum FGF21 and FGF19 are closely associated to hepatic Klotho expression in NAFLD. Importantly, it seems plausible that FGFs and/or their coreceptor mutually control their expression by direct feedback or by means of a common regulator of their tissue-specific promoters. For instance, as reported in **Figure S4**, the promoters of FGF21, FGF19 and Klotho coreceptor present binding regions for analogous transcription factors. Additional studies are required to identify potential NAFLD-activated molecular pathways involved in the down-regulation of FGF21-FGF19/Klotho network and its potential role in NAFLD-related progression, i.e. NASH, fibrosis and HCC.

In conclusion, our pediatric study shows a strong inverse association between FGF21 and FGF19 circulating levels, hepatic Klotho expression and the NAFLD severity suggesting a potential role for FGFs in the pathogenesis of NAFLD. If such findings will be replicated in external series of both pediatric and adult patients, this might have implications not only for a better understanding of the progression of NAFLD but also for the developments of novel targeted therapies.

## Supporting Information

Figure S1 **Control of secondary antibodies in control liver tissue.** Staining with (**A**) 1∶500 Alexa Fluor 488 goat anti-rabbit IgG, (**B**) Alexa Fluor 555 goat anti-mouse IgG secondary antibodies and, (**C**) both with DAPI. The white bar represents a 30 µm length.
(DOC)

Figure S2 **Positive controls for Klotho staining.** Klotho expression in gut (**A**) and kidney (**B**). Nuclear staining with DAPI. The white bar represents a 30 µm length.
(DOC)

Figure S3 **Klotho regions and forms.** Klotho protein contains KL1 and KL2 that are two repeat sequences in extracellular region, one transmembrane region and one small of cytoplasmic region. The types of secreted and cytoplasmic forms generated by Klotho cleavage remains to be fully characterized. Our antibody specifically recognizes a C-terminus sequence within the cytoplasmic region.
(DOC)

Figure S4 **Promoter sequences obtained from Eukariotic Promoter Database (** http://epd.vital-it.ch **) and Jaspar transcription factor binding sites obtained with JASPAR database for Vertebrata (** http://jaspar.binf.ku.dk/ **).** (**A**) Promoter region (−1000→1) for FGF21 human gene (ENSEMBL Gene ID: ENSG00000105550); (**B**) Promoter region (−1000→1) for FGF19 human gene (ENSEMBL Gene ID: ENSG00000162344); (**C**) Promoter region (−1000→1) for Klotho human gene (ENSEMBL Gene ID: ENSG00000133116). The putative sites in each sequence were predicted with 80% score threshold, only some of the common factors with score >7 were highlighted (see legend). Blue arrow indicates the transcription start site.
(DOC)
