# Supplementary figures and images for: Association between Serum Atypical Fibroblast Growth Factors 21 and 19 and Pediatric Nonalcoholic Fatty Liver Disease
Source: PLoS One. 2013 Jun 26;8(6):e67160. doi: 10.1371/journal.pone.0067160 (PMC3694051; doi:10.1371/journal.pone.0067160)

**
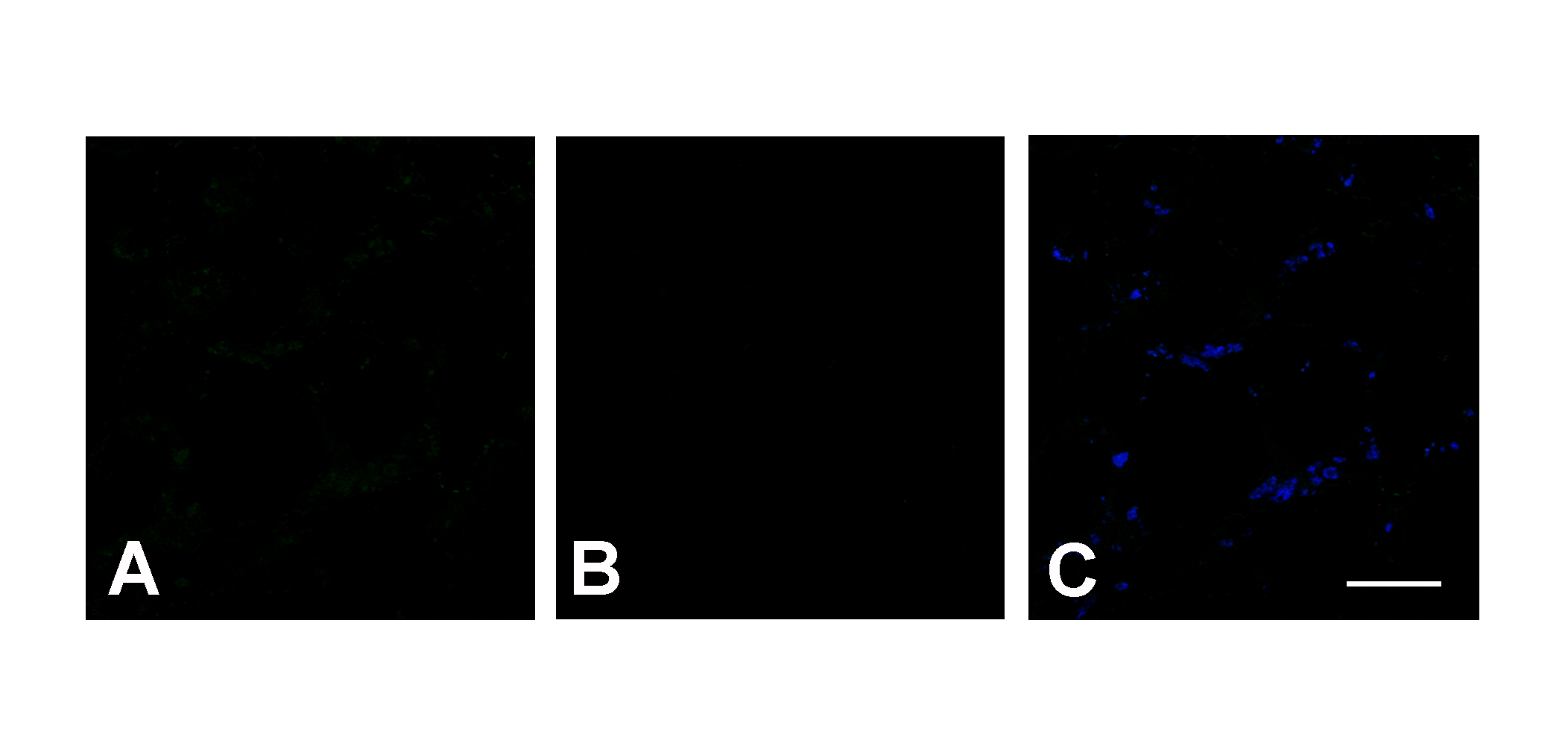
**

Supplement: Figure S1 — Control of secondary antibodies in control liver tissue. Staining with (A) 1∶500 Alexa Fluor 488 goat anti-rabbit IgG, (B) Alexa Fluor 555 goat anti-mouse IgG secondary antibodies and, (C) both with DAPI. The white bar represents a 30 µm length. (DOC) [file pone.0067160.s001.doc]

**
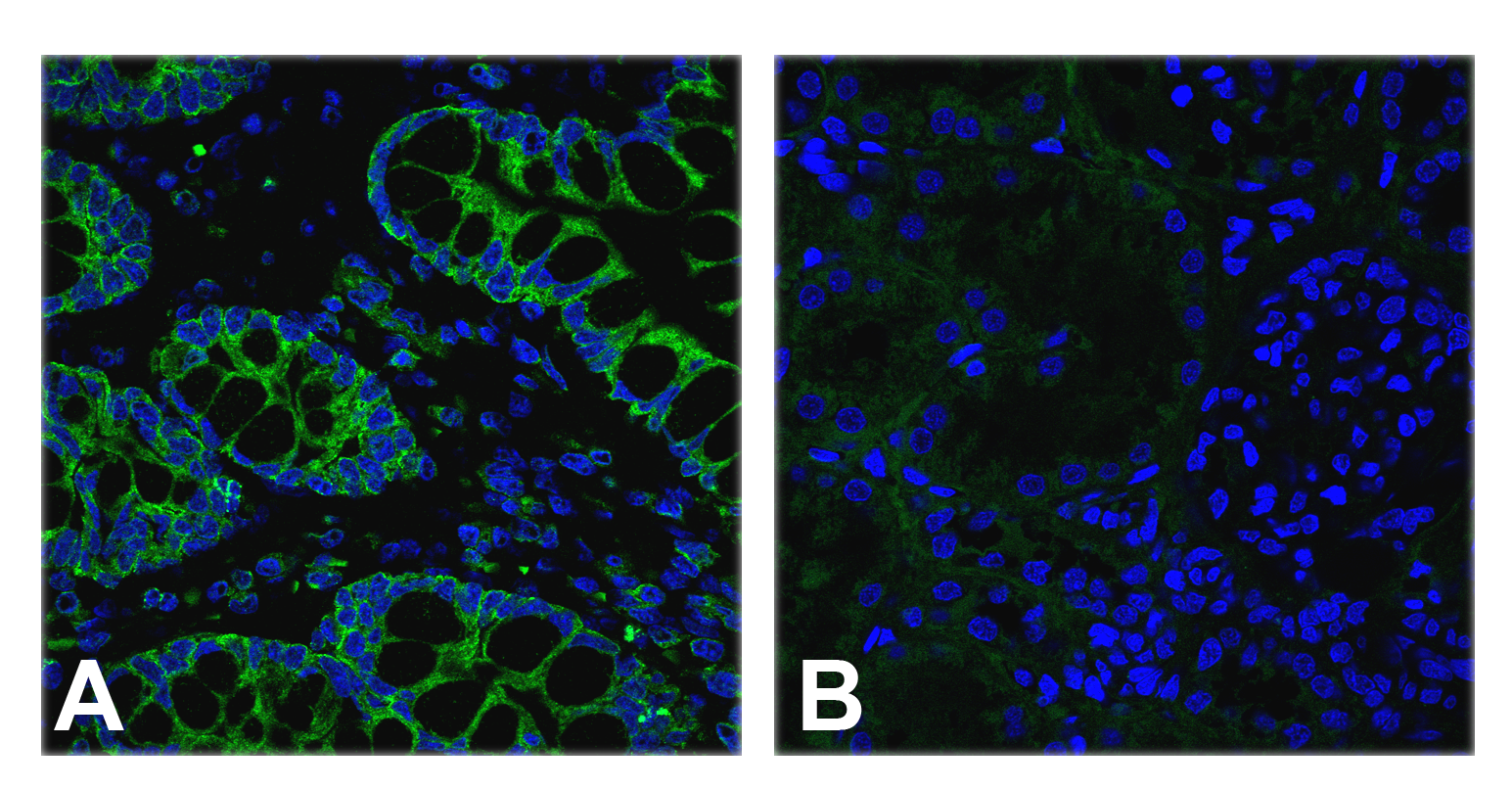
**

Supplement: Figure S2 — Positive controls for Klotho staining. Klotho expression in gut (A) and kidney (B). Nuclear staining with DAPI. The white bar represents a 30 µm length. (DOC) [file pone.0067160.s002.doc]

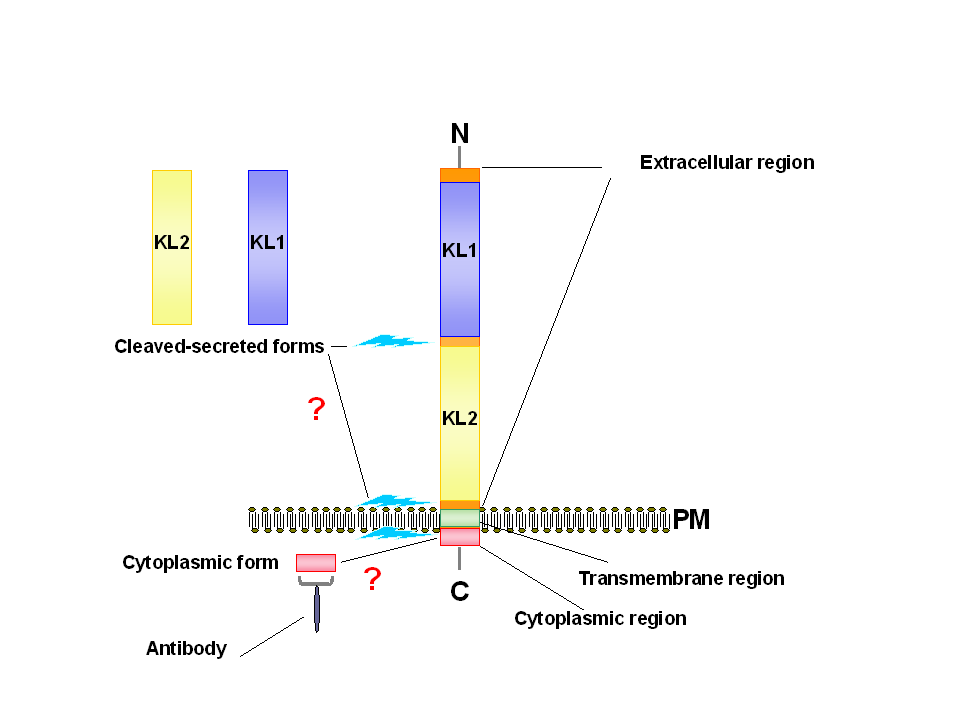

Supplement: Figure S3 — Klotho regions and forms. Klotho protein contains KL1 and KL2 that are two repeat sequences in extracellular region, one transmembrane region and one small of cytoplasmic region. The types of secreted and cytoplasmic forms generated by Klotho cleavage remains to be fully characterized. Our antibody specifically recognizes a C-terminus sequence within the cytoplasmic region. (DOC) [file pone.0067160.s003.doc]

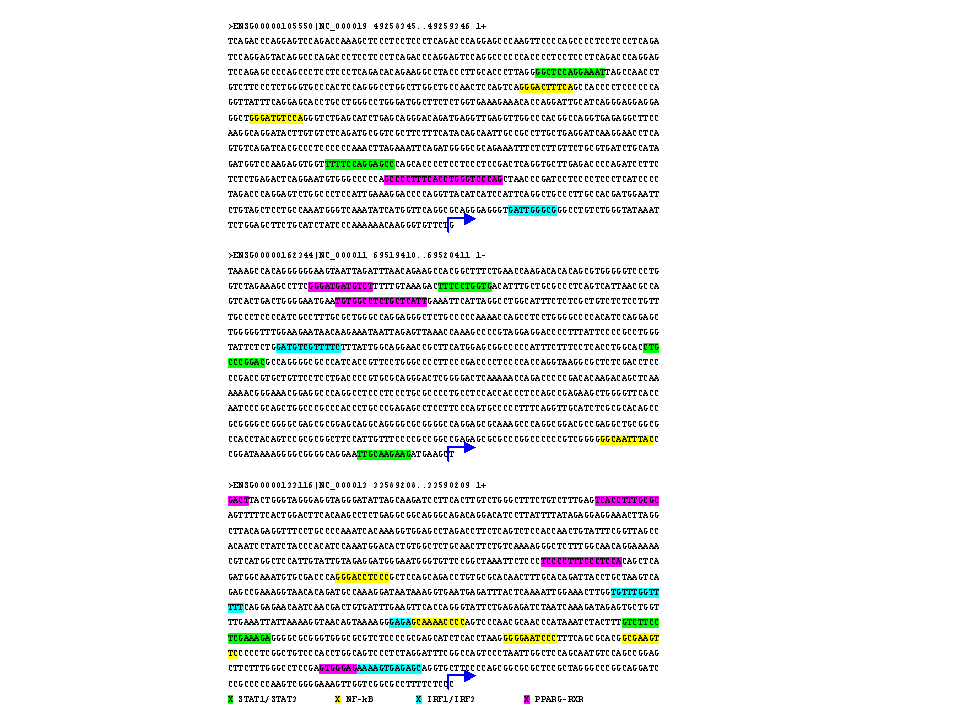

Supplement: Figure S4 — Promoter sequences obtained from Eukariotic Promoter Database ( http://epd.vital-it.ch ) and Jaspar transcription factor binding sites obtained with JASPAR database for Vertebrata ( http://jaspar.binf.ku.dk/ ). (A) Promoter region (−1000→1) for FGF21 human gene (ENSEMBL Gene ID: ENSG00000105550); (B) Promoter region (−1000→1) for FGF19 human gene (ENSEMBL Gene ID: ENSG00000162344); (C) Promoter region (−1000→1) for Klotho human gene (ENSEMBL Gene ID: ENSG00000133116). The putative sites in each sequence were predicted with 80% score threshold, only some of the common factors with score >7 were highlighted (see legend). Blue arrow indicates the transcription start site. (DOC) [file pone.0067160.s004.doc]
